# Do Age and Educational Stage Influence No-Mobile-Phone Phobia?

**DOI:** 10.3390/ijerph18094450

**Published:** 2021-04-22

**Authors:** Antonio-Manuel Rodríguez-García, José-Antonio Marín-Marín, Juan-Antonio López-Núñez, Antonio-José Moreno-Guerrero

**Affiliations:** 1 Department of Didactics and School Organization, University of Granada, 52071 Melilla, Spain; arodrigu@ugr.es; 2 Department of Didactics and School Organization, University of Granada, 18071 Granada, Spain; juanlope@ugr.es; 3 Department of Didactics and School Organization, University of Granada, 51001 Ceuta, Spain; ajmoreno@ugr.es

**Keywords:** nomophobia, smartphone, addictions, teenagers, youth

## Abstract

Technological progress not only brings with it resources that improve and facilitate the day-to-day life of the people who make up society but also entails health risks, with the emergence of terms, such as nomophobia, which is considered an anxiety disorder produced by the fear that not having a mobile phone generates in a person. This research aims to identify the relationship and influence between levels of nomophobia and the age or educational stage of students. The research method is based on a correlational and predictive design of quantitative methodology. The instrument used is the NMP-Q questionnaire. The study population is students from different educational stages (obligatory secondary education, baccalaureate, vocational training and university). The results show that students over 12 years old present an average level of “nomophobia” (no-mobile-phone phobia), namely, not being able to communicate with the family where the highest levels are presented. We conclude that students over 12 years of age and of any educational stage present an average level of nomophobia, and it cannot be determined that either the educational stage or the age are determining factors in the presentation of this problem. This can occur at any age and at any level of the different educational stages, although there are risk indicators that we should bear in mind to avoid the appearance of nomophobia.

## 1. Introduction

Today, technology, specifically mobile devices, is fully immersed in our society. One example of this is the smartphone, which is used and used today by practically the entire population, from children to adults, to access mainly information [1] and to be able to communicate with others [2]. We are probably looking at a device whose characteristics—its portability, ubiquity and immediate access—allow society to access the Internet from anywhere and at any time [3], being with it 24 h a day, 7 days a week [4], thereby provoking new habits and actions on the part of people in their daily lives [5].

These changes in daily life are generating new behaviors by people, leading to technological dependence [6], addiction [7] and disruptive behaviors [8,9]. All of this has led to the coinage of a new term, called “nomophobia” (no-mobile-phone phobia), which is defined as an anxiety disorder produced by the fear of being unable to access one’s mobile phone, either because it has been forgotten at home or because it is not operational [10].

Phobias are associated with frequent anxiety disorders. In addition, they are often associated with psychiatric disorders, such as depression or drug use [3]. Nomophobia is directly related to the misuse of Internet users [5]. This is due to excessive time spent on various online actions, generating problems in the individual, such as fears, conflicts with oneself or with others, or the impossibility of disconnecting, among others [9].

This leads to a dependence on different technological devices, including mobile phones [11]. In this case, nomophobia does not focus on the lack of control over using the mobile phone but on the pathological fear generated by its lack of use [6].

Therefore, it can be stated that nomophobia is considered as a specific anxiety disorder that encompasses various symptoms and behaviors, such as obsession, anxiety or the environment [12]. This is generated due to the inability to not visualize the mobile phone screen to see if you have received messages or emails, the fact of running out of battery, not being able to contact family or friends, among others [13].

The authors [8] mention that the clinical characteristics of nomophobia are the impulsive, excessive and uncontrolled use of technological devices. Other authors add other manifestations to characterize this syndrome [11,12]: always carrying the device with them and spending considerable time on it, always carrying a charger with them to avoid disconnection and even having several devices within reach.

These characteristics were delimited into four dimensions that would explain nomophobia [13]: fear of not being able to communicate with other people instantly; loss of connection; inability to access information immediately; and discomfort due to not having the mobile device with us.

Although current research is still in its early stages [12], this problem seems to affect more young people, especially those between 14 and 16 years old and women [14,15,16,17,18,19,20,21]. However, other research has not found significant data in this regard [22,23]. Therefore, state that this problem can be present at any age.

More specifically, the study by [14] found a high prevalence of nomophobia among students, who were more afraid of not being able to access information. Moreover, the younger the age of the student, the higher the level of nomophobia. In this case, women had higher levels of nomophobia than men due to their gender’s use of the telephone. These authors argue that men use it more for work, and women use it more to access social networks. In the study by [16], they showed that at younger ages, students felt higher levels of nomophobia, mainly focused on discomfort, anxiety, anger and insecurity about not using a mobile phone. In research [17] showed that females had higher levels of nomophobia than males. In addition, the older the age of the participants, the lower the levels of nomophobia. In addition, the greater using the telephone, the greater the likelihood of becoming nomophobia. Research [20] shows that the younger the age, the higher the likelihood of nomophobia. The research [21] reconfirms what was previously established, in that nomophobia was significantly related to gender, age group and level of education, and frequency of smartphone use was significantly related to age group and level of education. There was a positive correlation coefficient between nomophobia and frequency of smartphone use. In this case, females and younger people had higher levels of nomophobia. In contrast, in research [22,23], there was no evidence that the age and gender of the individual influenced levels of nomophobia, although it was shown to be significant that the higher the usage, the higher the level of nomophobia. Thus, most studies show that the age and gender of students are factors influencing levels of nomophobia.

Nevertheless, it seems that nomophobia is becoming one of the emerging diseases of the 21st-century around the world [12]. However, scientific production in Spain is still insufficient to draw any convincing conclusions between nomophobia, age and the educational stage. Given these circumstances, this work is based on the following research hypotheses:

- H1: Nomophobia will be higher in younger people;
- H2: Nomophobia will be higher in people at earlier educational stages.

For corroboration or refusal, this research arises to identify the relationship and influence between nomophobia and these two variables (age and educational stage) of the students. From this general objective, the following specific objectives arise:

- To identify the relationship and effect of students’ age and levels of nomophobia.
- To find out the correlation and effect of educational stage and levels of nomophobia.

## 2. Materials and Methods

The study is based on a transversal and analytical design based on a quantitative methodology [24].

### 2.1. Sample

The research was carried out with students from Ceuta, which is a city located in the south of Spain. The students belong to different educational stages, including higher education, secondary education, vocational training and baccalaureate. The sampling technique applied was simple random sampling, with a total population of 13,721 students. In this case, the sample is probabilistic, as all students had the same chance of being chosen. In this case, the first step was to calculate the representative size of the population. For this, an estimated percentage of 50% was applied, with a confidence level of 99% and a margin of error of 3%. Subsequently, the sample elements were selected, trying to ensure that all had the same chance of being chosen at the outset. To do this, we applied for the random numbers, as described in the appendix of [24]. The final sample is made up of 1630 students, 931 women (57.1%) and 699 men (42.9%). The participants are aged from 12–14 years (31.7%), 15–17 years (43.3%), 18–20 years (10.4%) and over 20 years (14.4%) and come from different educational stages of the Spanish educational system: bachelor’s degree (9.4%), vocational training (6.4%), compulsory secondary education (50.9%), high school (27%) and master’s degree (6.4%).

### 2.2. Instrument

The nomophobia questionnaire (NMP-Q) instrument adapted to the Spanish context was used to obtain the data [18,25]. The instrument has four dimensions, with a total of 20 items. In addition, a socio-educational dimension was used, consisting of 7 items.

The items of the four dimensions have a 7-point Likert scale. The minimum scores of the instrument can be 20, showing high levels of nomophobia. The maximum scores can be 140, showing very low levels of nomophobia. For the socio-educational dimension, other Likert scales and dichotomous questions have been used.

The validity and reliability of the questionnaire, developed by [25], presents statistical data that shows that it is a valid instrument, as demonstrated by the results obtained in the Keiser–Meyer–Olkin statistic (0.90) and the Bartlett test (x_2190_ = 1420.8259, *P* < 1). It also presents a variance greater than 62.7% in the four dimensions with initial values greater than 1, a variance in the second analysis by varimax rotation. Dimension I showed a variance of 22.38%, dimension II a variance of 16.82%, dimension III a variance of 11.87% and dimension IV a variance of 11.59%. It is also considered reliable since the value obtained in Cronbach’s alpha is 0.928, which shows good internal consistency.

### 2.3. Study Variables

The study variables were distributed between independent and dependent variables, establishing a codification for each one of them to facilitate their presentation and understanding of the results. The independent variables are based on the items obtained from the NMP-Q questionnaire. These variables are specified and coded in Table A1.

As dependent variables, we have considered “the age of the students” (E) and “educational stage” (ED). Equitable groups established the age ranges. We tried to group the age in groups of three, trying to have an equitable criterion in the distribution of years. In addition, we have established age groups different from those established in general for the educational stages themselves to avoid showing redundant and repetitive information.

The fact of establishing the NMP-Q variables as independent variables and the sociodemographic variables of age and educational stage as dependent variables is because of knowing the effect of nomophobia levels on age and educational stage. To this end, the premises of [24] were taken into account.

### 2.4. Procedure

The study presented here began with an exhaustive search of the scientific literature on the subject under investigation. Once the instrument was determined, we proceeded to establish contact with the teachers, who carry out their educational practice in the stages of compulsory secondary education, baccalaureate, vocational training and university education, requesting their collaboration. Permission was requested beforehand from the different schools and the request for data on the study population. At all times, all the appropriate steps were followed for applying for permissions in a formal manner, using a written document endorsed by the research group.

It should be noted that all participants, both students and schools, did so on a voluntary basis. In this case, it should be noted that the students who were selected after applying the sampling technique explained above agreed to participate. There were no students selected who refused to participate.

In addition, the students were informed of the study that was to be carried out. Those who were minors had to submit a consent form signed by the student’s legal guardians.

The questionnaire was then transcribed by selecting the Google form to facilitate data collection from all students participating in the study at all times. Participation was voluntary and anonymous, trying to achieve sincerity in the responses of those involved in the study. The participating students were participative. Data collection was carried out at the schools. None of the participants had any problems or difficulties in answering the instrument. Moreover, none of them had difficulties in handling the technological device used to collect the data.

It should be noted that the researchers carried out the data collection. They were present at all times in the schools, which provided their computer equipment to facilitate data collection.

### 2.5. Data Analysis

The statistical analysis was carried out with the SPSS v.25 statistical software (IBM, New York, United State). First, several tests were applied to determine whether or not parametric statistics were used. The first analyses showed that the various assumptions established for using parametric tests were not violated. It was, therefore, decided in this research to apply parametric tests [26,27].

For the statistical study, we performed a descriptive analysis of the means obtained and the standard deviation obtained for each of the nomophobia variables concerning age and educational stage. Pearson’s bivariate correlations were also applied, checking whether the linear association between age and educational stage concerning the nomophobia questionnaire variables, to identify whether they are statistically significant, as well as their strength and direction. Finally, multiple linear regression was applied by the stepwise method, which informs us about the dependence between the variables, trying to find out to what extent the nomophobia variables can be explained by the age of the students and the educational stage [28].

## 3. Results

Beginning with the description of the results obtained, it can be observed, in general terms, that the means obtained for each of the independent variables in relation to the variables age and educational stage are situated at levels between 3 and 5, placing the levels of nomophobia in a medium zone. Below the mean of 3 are several combinations of variables, including the combination of the variable NMF_2 concerning the age of the participants between 12 and 14 years old and the students in the baccalaureate stage. In addition, the combination established between the variable NMF_1 with the stage of Vocational Training. In all these combinations, it can be determined that the levels of nomophobia are high.

On the other hand, above the mean of 5 is the combination between the variable NMF_10 and the age between 15 and 17 years or the baccalaureate stage. Or the combination where the highest relationship is established, which is the case of the variable MFN_17 with age between 18 and 20, age over 20, students in the vocational training stage, bachelor’s degree students and master’s degree students. In all these combinations, the levels of nomophobia are low.

Going into more depth, the means presented for each of the independent variables concerning the two dependent variables show even means, which do not exceed. Only one exception is observed, that between the variable NMF_1 and the educational stage, where there is a significant contrast between the vocational training stage and the rest of the educational stages (Table 1).

The bivariate correlations established between the independent variables concerning the dependent variables show disparate levels of significance, given that not all correlations are significant. In addition, the strength of the relationship is low in all cases, below 0.02, which suggests the low influence of the dependent variable on the independent variable (Table 2).

In the two established multiple linear regression models, we have attempted to understand the effect of nomophobia on age and educational stage, respectively. In the first regression, in the marked connection between age and nomophobia, 7 models have been thrown, being the last one the one that throws a greater value of R2, the age could be explained by a 0.4% of the variance using the variables NMF_18, NMF_16, NMF_3, NMF_11, NMF_2, NMF_17 y NMF_14. In the second regression, three models were obtained, the last one giving a higher value in R2, with the educational stage being explained by 0.1% of the variance using the variables NMF_7, NMF_3 y NMF_2. Although in both cases, the values of R2 are low, they show a significant trend, allowing us to know the variables of nomophobia that influence age and educational stage (Table 3).

The variables that explain the multiple linear regression model, both in relation to age and educational stage, give values in B below 0.1, with the relationship established in some cases as negative, which shows an inverse proportional relationship. According to the values shown by t, which confirms the null hypothesis, it can be determined that the variables collected in Table 4 facilitate the explanation of the variance of the dependent variable. In this case, these are those that offer a greater predictive capacity.

## 4. Conclusions

Nomophobia is a topic of growing interest on which there is still much research to be done [12]. However, the findings found so far indicate that we are dealing with a pathology caused by the irrational and uncontrolled use of mobile devices. Such are the risks of nomophobia that certain sectors of society are beginning to indicate that we are facing a possible public health problem [8,29], being considered a disease of the 21st century [30,31,32], which in many cases requires drugs and psychological treatment to minimize the effects of addiction, and in the best of cases, to root it out [5]. Even today, there have been cases in which nomophobia causes physical problems [33], such as carpal tunnel syndrome [34], or physiological problems [35], focusing on sleep disorders and lack of rest [36].

The person’s profile with nomophobia appears to be a young person between 14 and 16 years old [18], although other research extends the age range to adolescents between 12 and 18 years old [25,37,38]. However, other research does not find significant data in this line [22,23]. Moreover, the educational stage also has been another variable studied by other research (high school, university, among others), such as [7,39].

The present study aimed to provide more data on the influence of age and educational stage on the presence of nomophobia in young students in the Autonomous City of Ceuta. It should be noted that this study was developed before the onset of the COVID-19 pandemic. As has been seen, in general terms, and for the context in which this research was carried out, the levels of nomophobia among students over 12 years of age are in an intermediate stratum, and there is no serious incidence of nomophobia. This differs from several research studies, where there seems to be agreement that nomophobia is more prevalent in younger people [14,15,16,17,18,19,20,21]. In other words, among students in the Autonomous City of Ceuta, younger students may not necessarily have high levels of nomophobia.

Analyses indicate that young people aged 12–14 show higher levels of nomophobia, especially in terms of not being able to communicate with their family instantly [15,16,17,18,19,20,21]. However, it appears that from the age of 15 years onwards, indicators of nomophobia begin to decline in the population studied. In other words, this does coincide with other previous studies, where the older the age, the lower the levels of nomophobia in the student population. Concerning the educational stage, the findings indicate that there is no dominant prevalence of nomophobia in any of the educational stages. It is, therefore, understood that the problem can occur at any of the stages studied [22,23] and is currently in an intermediate position. There is no defined prevalence for a specific age group, although it is true that the older the age, the lower the level of nomophobia.

Therefore, based on the results found, we can determine that there is no strong and consistent relationship between the items that can generate nomophobia and the age of the students or the educational stage at which they are studying. In those values in which there is a correlation, the strength of the association is very low, which confirms what has been indicated by [11], where they state that any age is susceptible to nomophobia. This confirms the findings of other studies [14,15,16,17,18,19,20,21], where levels of nomophobia are more closely associated with mobile phone use than with the age or educational stage of the students themselves.

However, on closer analysis, concerning age, running out of data signal to connect to a Wi-Fi network or running out of battery. Furthermore, not being able to receive text messages or calls again or not knowing what to do in case they run out of their mobile phone. In other cases, not knowing that the student’s family wants to contact them or running out of data and not being able to see notifications are risk factors for nomophobia. As pointed out by [13], we are dealing with symptoms related especially to not being able to communicate, not being able to access information and renunciation of comfort. In other words, in this case, to establish actions to reduce the levels of nomophobia, more attention should be paid to the risk factors mentioned above than to the age or educational stage of the subjects themselves.

We conclude that students over 12 years of age and of any educational stage present a moderate level of nomophobia. Therefore, we reject our initial hypotheses. It cannot be pointed out that the educational stage and age are determining factors in the presentation of this pathology, which can occur at any age and at any level of the different educational stages. However, there are risk indicators that we must take into account to avoid the appearance of nomophobia.

All of the above is triggering alterations in the affective-social development of children and adolescents [40], focusing on low self-esteem and high levels of sadness [41,42] that cause emotional instability [11] and life dissatisfaction [43], leading to depressive moods, aggressive behaviors, anger, anxiety [16], stress, restlessness and nervousness [18]. Such is the magnitude of nomophobia that students’ academic performance, attention levels, and degree of learning are being affected [7,44]. For this reason, from an early age, and especially in the stage of adolescence, training prevention activities should be carried out in relation to the responsible use of mobile devices.

The study’s limitations have focused on the procedure followed to start the research and data collection, given that in certain educational centers, teachers have not required permission from their management to pass the questionnaires, while in other centers, permissions have had to be formally presented. It was also difficult to have the computer room available for students to fill out the questionnaire, given that in the educational centers, said rooms are very limited and usually during most of the school hours. In addition, data collection was carried out during one week, which generated a situation of stress among the researchers. They had to give the appropriate guidelines to the different collaborators to adequately carry out all the established procedures. As a future line of research, we propose to analyze whether the socioeconomic and cultural level of the students is a factor that influences developing nomophobia.

## Figures and Tables

**Table 1:** Descriptive of averages by age and educational stage.

Likert Scale M/SD									
	Age				Educational Stage				
	12–14	15–17	18–20	+20	CSE	VT	BA	HS	MA
NMF_1	3.21/2.10	3.13/1.92	3.02/1.85	3.58/1.90	3.19/2.06	1.84/1.84	3.06/1.87	3.48/1.98	3.53/1.80
NMF_2	2.99/2.12	3.02/1.92	3.02/1.93	3.45/1.88	3.00/2.07	3.29/1.91	2.93/1.83	3.46/1.95	3.46/1.91
NMF_3	4.34/2.33	4.15/2.03	4.00/2.02	4.11/2.01	4.25/2.26	4.50/2.16	4.12/1.93	3.93/1.96	4.13/1.95
NMF_4	3.42/2.19	3.24/1.93	3.12/1.92	3.56/1.84	3.35/2.11	3.49/1.93	3.18/1.88	3.37/1.90	3.61/1.81
NMF_5	3.98/2.21	3.85/1.96	3.75/1.92	4.00/1.86	3.93/2.15	4.04/2.08	3.85/1.87	3.69/1.80	4.13/1.84
NMF_6	3.70/2.17	3.64/1.88	3.59/1.90	3.99/1.82	3.64/2.10	3.86/1.88	3.64/1.84	3.82/1.82	4.19/1.76
NMF_7	4.62/2.22	4.66/1.95	4.56/2.04	4.87/2.03	4.52/2.16	4.76/2.12	4.83/1.86	4.73/2.05	4.98/1.98
NMF_8	4.46/2.28	4.36/2.01	4.32/2.03	4.65/2.03	4.37/2.21	4.67/2.26	4.36/1.92	4.62/1.99	4.67/1.98
NMF_9	4.48/2.21	4.39/1.92	4.34/1.96	4.78/2.05	4.38/2.16	4.56/2.15	4.42/1.80	4.75/1.98	4.88/2.02
NMF_10	4.75/2.27	5.13/2.01	4.85/2.04	4.57/2.01	4.74/2.26	4.91/2.07	5.34/1.80	4.90/2.01	4.34/1.96
NMF_11	4.32/2.27	4.25/3.03	4.45/2.07	4.78/1.95	4.26/2.22	4.53/2.16	4.40/1.98	4.44/2.04	4.84/1.74
NMF_12	4.12/2.18	3.92/1.87	4.06/1.82	4.40/1.87	4.03/2.12	4.33/2.06	3.95/1.75	4.32/1.79	4.27/1.79
NMF_13	3.66/2.10	3.63/1.92	3.52/1.81	4.07/1.95	3.64/2.08	4.06/2.06	3.54/1.79	4.04/1.86	3.85/1.88
NMF_14	4.49/2.20	4.50/1.97	4.24/1.98	4.41/1.93	4.42/2.15	4.76/2.06	4.45/1.89	4.61/1.91	4.27/1.93
NMF_15	3.77/2.19	3.65/1.95	3.86/1.81	4.16/1.94	3.72/2.13	3.89/2.13	3.61/1.83	4.35/1.87	4.01/1.83
NMF_16	4.94/2.26	4.71/2.06	4.52/2.15	4.72/1.98	4.81/2.24	4.74/2.23	4.75/1.98	4.65/2.02	4.73/1.88
NMF_17	4.80/2.32	4.92/2.08	5.04/2.06	5.09/2.03	4.78/2.27	5.18/2.19	4.99/2.01	5.07/2.02	5.23/1.88
NMF_18	3.86/2.29	3.79/2.07	4.02/2.06	4.48/2.03	3.81/2.23	4.43/2.26	3.83/2.01	4.36/2.01	4.24/1.95
NMF_19	3.87/2.30	3.70/2.11	3.74/2.09	3.98/2.10	3.82/2.26	4.06/2.09	3.64/2.06	3.97/2.10	3.79/2.01
NMF_20	4.14/2.23	3.91/1.99	4.04/1.92	4.37/1.95	4.04/2.19	4.40/2.21	3.95/1.88	4.23/1.91	4.11/1.76

Note: M = mean; SD = standard deviation; CSE = compulsory secondary education; VT = vocational training; BA = bachelor’s degree; HS = high school; MA= master’s degree.

**Table 2:** Correlations between nomophobia variables with age and educational stage.

	Age		Educational Stage	
	*p*-Value	r	*p*-Value	r
NMF_1	0.072	0.045	0.205	0.031
NMF_2	0.010	0.064	0.026	0.055
NMF_3	0.081	−0.043	0.117	−0.039
NMF_4	0.763	0.007	0.900	0.003
NMF_5	0.822	−0.006	0.713	−0.009
NMF_6	0.140	0.037	0.040	0.051
NMF_7	0.202	0.032	0.004	0.070
NMF_8	0.042	0.020	0.0162	0.035
NMF_9	0.151	0.036	0.014	0.061
NMF_10	0.329	−0.024	0.183	0.033
NMF_11	0.006	0.068	0.013	0.061
NMF_12	0.107	0.040	0.272	0.027
NMF_13	0.037	0.052	0.203	0.032
NMF_14	0.348	−0.023	0.866	0.004
NMF_15	0.019	0.058	0.043	0.050
NMF_16	0.077	−0.044	0.430	−0.020
NMF_17	0.058	0.047	0.011	0.063
NMF_18	0.000	0.089	0.013	0.062
NMF_19	0.650	0.011	0.760	−0.008
NMF_20	0.219	0.030	0.787	0.007

Note: r = relationship strength.

**Table 3:** Multiple-step regression model.

**Age × Nomophofia (A × N)**				**Exchange Rate Statistics**			
**Model**	**R**	**R^2^**	**R^2^C**	**TEE**	**CR^2^**	**CF**	**SCF**
1	0.089	0.008	0.007	0.993	0.008	12.298	0.000
2	0.135	0.018	0.017	0.988	0.010	16.987	0.000
3	0.151	0.023	0.021	0.986	0.005	7.844	0.005
4	0.173	0.030	0.027	0.983	0.007	11.777	0.001
5	0.183	0.033	0.030	0.981	0.003	5.868	0.016
6	0.193	0.037	0.034	0.980	0.004	6.541	0.011
7	0.199	0.040	0.036	0.979	0.002	4.005	0.044
**Educational Stage × Nomophobia (ES × N)**				**Exchange Rate Statistics**			
**Model**	**R**	**R^2^**	**R^2^C**	**TEE**	**CR^2^**	**CF**	**SCF**
1	0.070	0.005	0.004	1.305	0.005	8.105	0.004
2	0.115	0.013	0.013	1.300	0.008	13.493	0.000
3	0.135	0.018	0.018	1.297	0.005	8.296	0.004

Note: R = R-statistic; R^2^ = R-squared; R^2^C = R-squared corrected; TEE = standard error of estimation; CR^2^ = change in R^2^; CF = change in F; SCF = significance change in F.

**Table 4:** Coefficients of the multiple linear regression model.

**E × N**	**B**	**Typical Error**	**Beta**	**t**	**Sig.**	**Tolerance**	**FIV**
7 (Constant)	1.995	0.078		25.510	0.000		
NMF_18	0.056	0.015	0.120	3.819	0.000	0.601	1.663
NMF_16	−0.068	0.016	−0.145	−4.320	0.000	0.529	1.890
NMF_3	−0.055	0.014	−0.118	−3.913	0.000	0.646	1.548
NMF_11	0.049	0.014	0.104	3.462	0.001	0.659	1.518
NMF_2	0.036	0.013	0.072	2.717	0.007	0.844	1.185
NMF_17	0.045	0.016	0.097	2.877	0.004	0.526	1.901
NMF_14	−0.028	0.014	−0.058	−2.014	0.044	0.703	1.422
**ED × N**	**B**	**Typical Error**	**Beta**	**t**	**Sig.**	**Tolerance**	**FIV**
3 (Constant)	1.956	0.089		21.897	0.000		
NMF_7	0.080	0.018	0.126	4.333	0.000	0.714	1.401
NMF_3	−0.081	0.019	0.132	−4.350	0.000	0.656	1.525
NMF_2	0.049	0.017	0.075	2.880	0.004	0.882	1.113

Note: B = B-static; t = *t*-static; Sig. = significance; FIV = collinearity statistic.

## Data Availability

Not applicable.

## Appendix A

The coding of each of the variables of the NMP-Q questionnaire is presented below.

**Table A1:** Coding of the variables of the NMP-Q questionnaire.

Code	Variable
NMF_1	I would be worried about not being able to communicate at the time with my family and/or friends
NMF_2	I would be worried about my family and/or friends not being able to contact me
NMF_3	I would be worried about not being able to receive text messages or calls
NMF_4	I would be worried about not being able to keep in touch with my family and/or friends
NMF_5	I would be nervous about not being able to know if someone had tried to contact me
NMF_6	I would be worried about not being in constant contact with my family and/or friends
NMF_7	I would be nervous about being disconnected from my virtual identity
NMF_8	I would feel bad about not being able to keep up with what is going on in the media and social networks
NMF_9	I would feel uncomfortable about not being able to check the notifications about my virtual connections and networks
NMF_10	I would be overwhelmed by not being able to check if I have new emails
NMF_11	I would feel weird because I wouldn’t know what to do
NMF_12	I would feel bad if I couldn’t access information on my smartphone at any time
NMF_13	I would be upset if I couldn’t check information on my smartphone when I wanted to
NMF_14	I would be nervous if I couldn’t access the news (p. I would be nervous if I couldn’t access the news (e.g., events, weather forecasts, etc.) through my smartphone
NMF_15	I would be upset if I couldn’t use my smartphone and/or its applications when I wanted to
NMF_16	I would be scared if my smartphone ran out of battery
NMF_17	I would be upset if I was about to run out of credit or reach my monthly spending limit
NMF_18	If I ran out of data signal or couldn’t connect to a Wi-Fi network, I’d be constantly checking to see if I got a signal or found a network
NMF_19	If I couldn’t use my smartphone, I’d be afraid I’d get stuck somewhere
NMF_20	If I couldn’t check my smartphone for a while, I’d feel like doing it

## References

[1] Kneidinger-Müller B. (2019). When The Smartphone Goes Offline: A Factorial Survey of Smartphone Users’ Experiences Of Mobile Unavailability. Comput. Hum. Behav..

[2] Moreno-Guerrero A.J., López-Belmonte J., Romero-Rodríguez J.M., Rodríguez-García A.M. (2020). Nomophobia: Impact of cell phone use and time to rest among teacher students. Heliyon.

[3] Lee S., Kim M., Mendoza J., McDonough I. (2018). Addicted To Cellphones: Exploring the Psychometric Properties Between the Nomophobia Questionnaire and Obsessiveness in College Students. Heliyon.

[4] Lin C., Griffiths M., Pakpour A. (2018). Psychometric Evaluation of Persian Nomophobia Questionnaire: Differential Item Functioning and Measurement Invariance Across Gender. J. Behav. Addict..

[5] Moreno-Guerrero A.J., Aznar-Díaz I., Cáceres-Reche P., Rodríguez-García A.M. (2020). Do Age, Gender and Poor Diet Influence the Higher Prevalence of Nomophobia among Young People?. Int. J. Environ. Res. Public Health.

[6] Santana-Vega L., Gómez-Muñoz A., Feliciano-García L. (2019). Adolescents Problematic Mobile Phone Use, Fear of Missing Out and Family Communication. Comunicar.

[7] Ahmed S., Pokhrel N., Roy S., Samuel A. (2019). Impact of Nomophobia: A Nondrug Addiction Among Students of Physiotherapy Course Using an Online Cross-Sectional Survey. Indian J. Psychiatry.

[8] Bragazzi N., Del Puente G. (2014). A Proposal for Including Nomophobia in the New DSM-V. Psychol. Res. Behav. Manag..

[9] Throuvala M.A., Griffiths M.D., Rennoldson M., Kuss D.J. (2019). Motivational Processes and Dysfunctional Mechanisms of Social Media Use Among Adolescents: A Qualitative Focus Group Study. Comput. Hum. Behav..

[10] Tangmunkongvorakul A., Musumari P., Thongpibul K., Srithanaviboonchai K., Techasrivichien T., Suguimoto S., Kihara M. (2019). Association of Excessive Smartphone Use With Psychological Well-Being Among University Students in Chiang Mai, Thailand. PLoS ONE.

[11] Argumosa-Villar L., Boada-Grau J., Vigil-Colet A. (2017). Exploratory Investigation of Theoretical Predictors of Nomophobia Using the Mobile Phone Involvement Questionnaire (MPIQ). J. Adolesc..

[12] Rodríguez-García A.-M., Moreno-Guerrero A.-J., López-Belmonte J. (2020). Nomophobia: An Individual’s Growing Fear of Being Without A Smartphone—A Systematic Literature Review. Int. J. Environ. Res. Public Health.

[13] Yildirim C., Correia A.-P. (2015). Exploring the Dimensions of Nomophobia: Development and Validation of a Self-Reported Questionnaire. Comput. Hum. Behav..

[14] Bhattacherjee S., Dasgupta P., Dasgupta S., Roy J., Mukherjee A., Biswas R. (2017). Nomophobic Behaviors Among Smartphone Using Medical and Engineering Students in Two Colleges of West Bengal. Indian J. Public Health.

[15] Bülbüloğlu S., Özdemir A., Kapıkıran G., Sarıtaş S. (2020). The effect of nomophobic behavior of nurses working at surgical clinics on time management and psychological well-being. J. Subst. Use.

[16] Darvishi M., Noori M., Nazer M., Sheikholeslami S., Karimi E. (2019). Investigating Different Dimensions of Nomophobia Among Medical Students: A Cross-Sectional Study. Open Access Maced. J. Med Sci..

[17] Gezgin D.M., Sumuer E., Arslan O., Yildirim S. (2017). Nomophobia Prevalence among Pre-service Teachers: A case of Trakya University. Trak. Üniversitesi Eğitim Fakültesi Derg..

[18] González-Cabrera J., León-Mejía A., Pérez-Sancho C., Calvete E. (2017). Adaptation of the Nomophobia Questionnaire (NMP-Q) to Spanish in a Sample of Adolescents. Actas Españolas De Psiquiatr..

[19] León-Mejía A., Calvete E., Patino-Alonso C., Machimbarrena J., González-Cabrera J. (2020). Cuestionario de Nomofobia (NMP-Q): Estructura factorial y puntos de corte de la versión española. Adicciones.

[20] Musa R., Saidon J., Rahman S.A. (2017). Who’s at Risk for Smartphone Nomophobia and Pathology, the Young or Matured Urban Millennials?. Adv. Sci. Lett..

[21] Soleymani M., Daei A., Ashrafi-rizi H. (2019). Nomophobia and health hazards: Smartphone use and addiction among university students. Int. J. Prev. Med..

[22] Jilisha G., Venkatachalam J., Menon V., Olickal J. (2019). Nomophobia: A Mixed-Methods Study on Prevalence, Associated Factors, and Perception among College Students in Puducherry, India. Indian J. Psychol. Med..

[23] Yildiz Durak H. (2019). Investigation of nomophobia and smartphone addiction predictors among adolescents in Turkey: Demographic variables and academic performance. Soc. Sci. J..

[24] Hernández Sampieri R., Fernández Collado C., Baptista Lucio M.P. (2016). Metodología de la Investigación.

[25] Gutiérrez-Puertas L., Márquez-Hernández V., Aguilera-Manrique G. (2016). Adaptation and Validation of the Spanish Version of the Nomophobia Questionnaire In Nursing Studies. Cin. Comput. Inform. Nurs..

[26] De la Fuente Sánchez D., Hernández Solís M., Pra Martos I. (2017). Vídeo Educativo Y Rendimiento Académico En La Enseñanza Superior a Distancia. Ried. Rev. Iberoam. De Educ. A Distancia.

[27] Montilla J.M., Kromrey J. (2010). Robustez De Las Pruebas T En comparación de medias, ante violación de supuestos de normalidad y homocedasticidad. Cienc. E Ing..

[28] Giner Mira I., Navas Martínez L., Holgado Tello F.P., Soriano Llorca J.A. (2019). Actividad física extraescolar, autoconcepto físico, orientaciones de meta y rendimiento académico. Rev. Psicol. Deporte.

[29] Basu S., Garg S., Singh M., Kohli C. (2018). Addiction-Like Behavior Associated With Mobile Phone Usage among Medical Students in Delhi. Indian J. Psychol. Med..

[30] Csibi S., Griffiths M.D., Cook B., Demetrovics Z., Szabo A. (2017). The Psychometric Properties of the Smartphone Application-Based Addiction Scale (SABAS). Int. J. Ment. Health Addict..

[31] Moreno-Guerrero A.J., Soler-Costa R., Marín-Marín J.A., López-Belmonte J. (2021). Flipped learning y buenas prácticas docentes en educación secundaria. Comunicar.

[32] Romero-Rodríguez J., Rodríguez-Jiménez C., Ramos Navas-Parejo M., Marín-Marín J., Gómez-García G. (2020). Use of Instagram by Pre-Service Teacher Education: Smartphone Habits and Dependency Factors. Int. J. Environ. Res. Public Health.

[33] Pivetta E., Harkin L., Billieux J., Kanjo E., Daria J.K. (2019). Problematic Smartphone Use: An Empirically Validated Model. Comput. Hum. Behav..

[34] Lee Y., Yang H., Jeong C., Yoo Y., Jeong G., Moon J., Hong S.W. (2012). Changes in the Thickness of Median Nerves Due to Excessive Use of Smartphones. J. Phys. Ther. Sci..

[35] Gentina E., Tang T.L.P., Dancoine P.-F. (2018). Does Gen Z’s Emotional Intelligence Promote Icheating (Cheating With Iphone) yet Curb Icheating through Reduced Nomophobia?. Comput. Educ..

[36] Panova T., Carbonell X., Chamarro A., Puerta-Cortés D.-X. (2019). Specific Smartphone Uses and How They Relate to Anxiety and Depression in University Students: A Cross-Cultural Perspective. Behav. Inf. Technol..

[37] Betoncu O., Ozdamli F. (2019). The Disease of 21St Century: Digital Disease. Tem J..

[38] Ramos-Soler I., López-Sánchez C., Quiles-Soler M.-C. (2017). Adaptación y validación de la escala de nomofobia de yildirim y correia en estudiantes españoles de la educación secundaria obligatoria. Health Addict./Salud Y Drog..

[39] Yildirim C., Sumuer E., Adnan M., Yildirim S. (2016). A growing fear. Inf. Dev..

[40] Oliveira T., Barreto L., El-Aouar W., Souza L., Pinheiro L. (2017). Where’s My Cell Phone? An Analysis of Nomophobia in the Organizational Environment. Rev. Adm. Empresas.

[41] Ozdemir B., Cakir O., Hussain I. (2018). Prevalence of Nomophobia among University Students: A Comparative Study of Pakistani and Turkish Undergraduate Students. Eurasia J. Math. Sci. Technol. Educ..

[42] Rai A., Kamath R., Kamath L., D’Souza B. (2019). Mobile Phone Dependency and Self-Esteem among Adolescents. Int. J. Mech. Eng. Technol..

[43] Samaha M., Hawi N.S. (2016). Relationships among Smartphone Addiction, Stress, Academic Performance, and Satisfaction with Life. Comput. Hum. Behav..

[44] Mendoza J.S., Pody B., Lee S., Kim M., McDonough I.M. (2018). The Effect of Cellphones on Attention and Learning: The Influences of Time, Distraction, and Nomophobia. Comput. Hum. Behav..

